# Evaluation of coupling coordination degree between tourism urbanization and ecosystem services in urban agglomerations in the yellow river basin

**DOI:** 10.1038/s41598-025-05455-6

**Published:** 2025-07-01

**Authors:** Kejun Wu, Aoxue Xing, Gang Wei, Haonan Xin, Yating Wei, Lihui Su, Jingbo Zhou

**Affiliations:** 1 Tourism College of Northwest Normal University, Anning East Road No.967, Anning District, Lanzhou, 730070 China; 2 Tourism Development Research Academy of Gansu, Lanzhou, 730070 China

**Keywords:** Tourism urbanization, Ecosystem service, Spatiotemporal coupling coordination, InVEST model, Yellow river basin (YRB), Environmental impact, Ecosystem services, Urban ecology

## Abstract

Understanding the interaction between ecosystem services (ESs) and tourism urbanization (TU) is essential for regional development and decision-making in urban agglomerations in the Yellow River Basin (YRB). Evaluating the coupling coordination degree (CCD) between ESs and TU is vital for regional development in the YRB. This study employs multi-year statistical data and spatial datasets—including land use, digital elevation models, meteorological data, and soil property data—to assess tourism urbanization using the entropy method. Concurrently, ESs, which encompass water yield, soil conservation, habitat quality, and carbon sequestration, are evaluated through the InVEST model. A spatiotemporal analysis of the YRB from 2000 to 2020 reveals two key trends: (1) the spatial polarization of tourism urbanization has intensified, with 68% of high-intensity clusters concentrated in eastern provinces (e.g., Shandong, Henan), sharply contrasting with the underdeveloped western regions (e.g., Qinghai, Ningxia); and (2) the coupling coordination degrees between TU and ESs have declined by 22–35% in ecologically fragile zones, driven by habitat fragmentation and carbon loss. Specifically, water yield and soil conservation have increased by 18% and 24%, respectively, while habitat quality and carbon sequestration have decreased by 14% and 11% in urbanizing areas, reflecting unsustainable trade-offs. The novel contribution of this study lies in establishing a basin-scale CCD framework for urban agglomerations, providing empirical evidence to reconcile tourism-driven growth with ecosystem resilience in the YRB. These findings underscore the urgent need for spatially adaptive governance to mitigate developmental imbalances. Future research should integrate high-resolution data and cultural ESs to address micro-scale complexities.

**Supplementary Information:**

The online version contains supplementary material available at 10.1038/s41598-025-05455-6.

## Introduction

The urbanization rate in China has increased from 19.4% in 1980 to 65.2% by 2022, making it the country with the fastest urbanization process in the world since the inception of the reform and opening-up policy in 1978^1^. The impact of this transition on Chinese economic and social structure has been significant^2^. Tourism urbanization (TU), driven by economic growth and population expansion, has emerged as a significant force in shaping regional development^3,4^. While it promotes economic diversification and urban expansion, it simultaneously exerts both positive and negative influences on ecosystem services, impacting biodiversity, carbon sequestration, and water resources^5^. Tourism urbanization facilitates the transformation of land use through the rapid expansion of infrastructure, leading to increased pressure on natural ecosystems^6,7^. However, the resulting ecological stress often disrupts the balance between urban expansion and ecosystem sustainability, triggering issues such as habitat fragmentation and environmental degradation^8^. Critically, existing studies predominantly address TU’s economic or ecological impacts in isolation^9^, leaving a gap in systemic frameworks to balance these dual objectives—particularly in ecologically vulnerable basins like the YRB. This study fills this void by developing a Coupling Coordination Degree (CCD) model that quantifies the dynamic equilibrium between TU and ecosystem services (ESs). This approach not only advances the ‘sustainable tourism’ paradigm^10^ but also operationalizes the UN SDGs by aligning urbanization pathways with ES resilience in the YRB, a cornerstone of China’s ecological security.

Ecosystem services (ESs) encompass the diverse products that natural ecosystems offer to humans as well as the regulatory and protective functions of ecosystems on the environment^11,12^. As a bridge and link connecting natural ecosystems and the socio-economic system, ESs have become essential indicators for assessing the sustainable high-quality development and health of ecosystems since the Millennium Ecosystem Assessment^13^. Nevertheless, preceding studies primarily concentrated on evaluating the value and provisioning capacity of ESs^14,15^. The balance and synergy of ESs, the heterogeneity of internal relationships in ESs, and factors influencing the alignment of ESs supply and demand have attracted widespread attention from scholars domestically as well as abroad at present^16,17^. Furthermore, the efficiency and intensity of resource utilization in different locations are mostly influenced by tourism urbanization, especially over-tourism in coastal and ecologically fragile areas, which exacerbates the spatiotemporal dislocation of ESs^18^.

The coupling coordination relationship between tourism urbanization and ecosystem services has progressively emerged as an academic focal point in recent years^19^. Coupling Coordination Degree (CCD) refers to the extent to which two or more systems achieve synergistic interactions through coordinated mechanisms during their mutual evolution^20,21^. The CCD model has been widely adopted to evaluate their interactive dynamics, providing critical theoretical foundations and practical guidance for regional sustainable development. In the context of tourism urbanization and ecosystem services, CCD quantifies the synergistic effects between tourism-driven economic development and ecological conservation. Studies demonstrate that a high CCD signifies efficient mutual promotion between tourism urbanization and ecosystem services, whereas a low CCD reflects contradictions or imbalances between these systems^22^.

Existing research on the coupling relationship between tourism urbanization and ecosystem services often focuses on individual regions^23–25^ or specific urban agglomerations^26,27^ rather than urban agglomerations in river basins. Urban agglomerations, as complex systems involving multiple interacting cities, require specific analysis to understand the integrated effects of tourism urbanization and ecosystem services. This study fills this gap by evaluating the coupling coordination degree (CCD) between TU and ES across urban agglomerations in the Yellow River Basin (YRB).

The impact mechanisms of tourism urbanization on ecosystem services have been explored in various regions, but findings are often context-dependent^28^. For instance, tourism urbanization accelerates the transformation of natural landscapes into urbanized areas, affecting water regulation, carbon storage, and habitat quality^29–31^. On one hand, the rapid conversion of natural ecosystems weakens ecosystem functions, disrupting biodiversity and increasing environmental risks such as soil erosion and desertification^32,33^. On the other hand, intensified tourism activities elevate the demand for ecosystem services, leading to spatial mismatches between supply and demand, particularly in ecologically fragile areas^34,35^.

In the Yellow River Basin, the unique ecological and geographical characteristics necessitate a more nuanced understanding of these impacts, which remains underexplored^36,37^. Due to regional variations in climate and socio-economic factors, the efficiency and intensity of resource utilization differ significantly across the basin. For example, the arid upstream Loess Plateau (annual precipitation < 400 mm)^38^ faces stringent water constraints that limit conventional tourism infrastructure^39^, while the flood-prone downstream Shandong Peninsula struggles with seasonal habitat degradation^40^. Socio-economically, high-income urban clusters like Zhengzhou harness advanced logistics networks^41^ to develop eco-tourism, whereas pastoral regions (e.g., Inner Mongolia) prioritize low-impact cultural tourism akin^42^ to the Yangtze Delta’s ‘green villages’^43^. These disparities underscore the need for adaptive strategies that reconcile TU growth with localized ecological thresholds—a gap this CCD model directly addresses.

The ecosystem of the YRB is fragile and highly susceptible to anthropogenic pressures, including tourism urbanization^44^. Despite its ecological significance, the interactions between tourism urbanization and ESs remain insufficiently understood, particularly regarding their spatial and temporal variations^45^. Most existing studies focus on single-factor analyses, overlooking the complex feedback loops between urbanization and ecosystem health. Moreover, systematic assessments of tourism-related land-use transformations and their long-term effects on ESs are lacking^46^. A more integrated approach is needed to capture the multidimensional impacts of tourism urbanization on ecosystem sustainability^15^.

This study aims to systematically assess the impact of tourism urbanization on ecosystem services in the YRB through spatiotemporal analysis, revealing its dynamic characteristics and impact mechanisms. The research addresses three core questions: (1) How has the coupling coordination degree between tourism urbanization and ecosystem services evolved spatially and temporally? (2) What are the key drivers influencing their interaction? (3) What policy implications can be derived for sustainable development?

The InVEST model and the Coupling Coordination Degrees (CCD) across the YRB provide a spatially explicit assessment of ecosystem service trade-offs and synergies. Additionally, ecosystem service valuation (ESV) is employed to evaluate both biophysical and socio-economic drivers of ES change^47^.

China’s national strategy, the “Ecological Protection and High-quality Development of the Yellow River Basin,” calls for a balanced approach to urbanization and ecological conservation. By addressing the spatiotemporal dynamics and impact mechanisms, this study offers practical insights for regional ecological management and sustainable tourism development. The findings provide a scientific basis for spatially adaptive governance and sustainable urban planning in the Yellow River Basin. This study directly informs policymakers by providing empirical evidence on how tourism urbanization can be harmonized with ecosystem services (ES) to achieve regional sustainability goals.

## Methods and data

### Research area

**Fig. 1:**
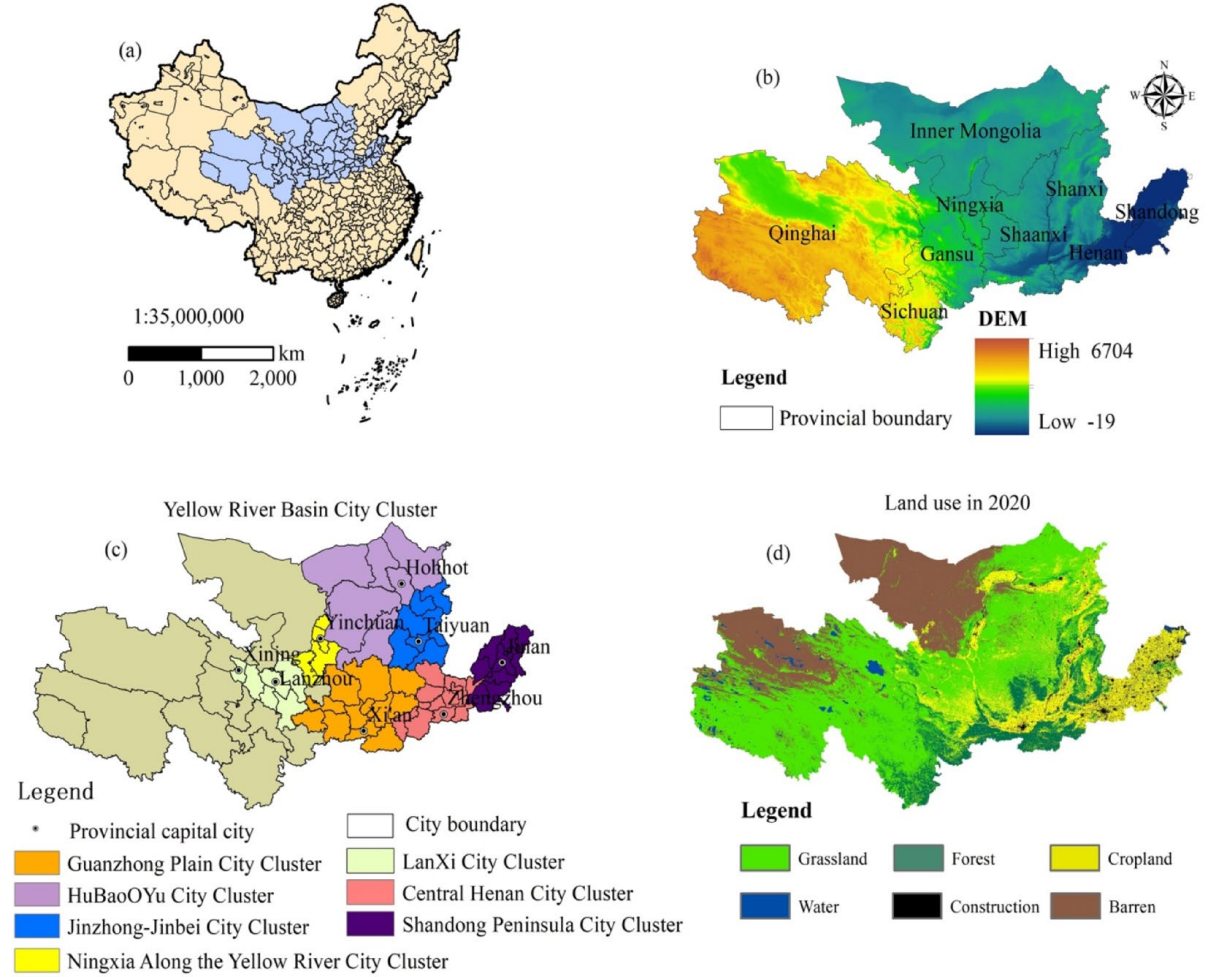
Geographical location, elevation, city clusters, and land use of the YRB.

The Yellow River Basin (YRB) (Fig. 1), spanning approximately 795,000 km^2^ across four geomorphic units (Qinghai-Tibet Plateau, Inner Mongolia Plateau, Loess Plateau, and North China Plain), is a critical ecological and economic corridor in China (Fig. 1a)^48^. Characterized by three distinct topographic terraces (Fig. 1b), this region includes the natural ecological corridor and several important functional ecological zones including the Ruoergai, Qilian Mountains and Three Rivers Source^46^.

*Note*(**a**) geographical location of YRB in China; (**b**) elevation; (**c**) Yellow River Basin city cluster; (**d**) Land use and cover in the study area in 2020, illustrating the different land use and cover including grassland, forest, cropland, water bodies, construction land, and barren land. All graphs presented in this study were created by the author using ArcGIS 10.8, developed by the Environmental Systems Research Institute (ESRI) (https://www.esri.com/).

As China’s second-largest economic belt, the YRB hosts seven national-level city clusters (Fig. 1c), including the Guanzhong Plain and Lan-Xi clusters, which collectively account for 63.3% urbanization rate and 412.9 billion CNY tourism revenue (2021)^46^.

The YRB exhibits significant climatic and land-use heterogeneity (Fig. 1d). (1) Climate: Annual precipitation ranges from 300 mm (northwest) to 600 mm (southeast), with 70% concentrated in July–August^29^. (2) Land use: Dominated by grassland (42%), cropland (28%), and forest (18%), while construction land (7%) is clustered in downstream urban clusters (e.g., Shandong Peninsula)^47^. Rapid urbanization and tourism expansion (projected 79% urbanization by 2035) intensify pressures on ecosystem services, particularly in ecologically fragile upper reaches with extensive barren land (Fig. 1d)^48^.

### Data sources

Two main forms of data are primarily utilized in this study, the first includes the integration of multi-year, municipal-scale statistical yearbook data from the YRB, used to evaluate the level of tourism urbanization advancement; Furthermore, this study evaluates the four types of ESs functions based on the multi-source and multi-scale spatial datasets, including land use, DEM, meteorological, soil attribute, and road data. For a more detailed overview of the data sources and data processing methodologies, refer to Table 1.

**Table 1:** Data for the analysis in study.

Data type	Source	Data description
Land use/Land cover	30 m annual land cover and its dynamics in China from 1990 to 2019 10.5281/zenodo.8176941	Raster(30 × 30 m) Reclassify Land Use Types as Cropland, Forest, Grassland, Water Bodies, Construction, and Barren
DEM data	Digital Elevation Model (DEM) http://www.gscloud.cn/	Raster (30 × 30 m). An elevation value for every grid cell.
Potential Evapotranspiration Data	National Tibetan Plateau Data Center 10.11866/db.loess.2021.001	Based on monthly average, minimum, and maximum temperature data, the Hargreaves method was used to calculate potential evapotranspiration.
Precipitation	National Tibetan Plateau Data Center 10.5281/zenodo.3185722.	The global 0.5° climate dataset by CRU and the global climate dataset released by WorldClim, using the Delta spatial downscaling scheme.
Soil Property data	Harmonized World Soil Database (HWSD V1.2) https://www.fao.org/	The characterization of soil parameters including organic carbon, water storage capacity, soil depth, textural class.
Statistical Yearbook	“China Urban Statistical Yearbook” “National Economic and Social Development Statistical Bulletin”,“China Tourism Yearbook”	The Development Level of the Secondary and Tertiary Industries and their Relationship with Employment, Infrastructure Construction, and Environmental Protection Efforts
Vector Data and Road Data	Resource and Environment Science and Data Center http://www.resdc.cn/DOI	Railway, Expressway, National Highway, and Provincial Highway Data Network

Note: This table summarizes the essential datasets utilized in the research, indicating the provenance, spatial resolution, and description for each data type. The data types include land use/land cover, Digital Elevation Model (DEM), potential evapotranspiration, precipitation, soil property data, statistical yearbooks, and vector and road data.

### Methods

#### Assessment of tourism urbanization

##### Composite index system

To evaluate tourism urbanization (TU) in the Yellow River Basin (YRB), this study developed a composite index system integrating three subsystems:

*Urbanization subsystem* Measures socio-economic progress through population urbanization (e.g., urban population density), infrastructure (e.g., per capita GDP), and resource management^49^.

*Tourism industry subsystem* Quantifies tourism’s economic impact via income (domestic/international), tourist arrivals, and infrastructure (e.g., hotel density)^50^.

*Eco-environment subsystem* Evaluates environmental quality using the Pressure-State-Response (PSR) model, including pollution emissions (pressure), green space coverage (state), and waste utilization (response)^51^.

##### Entropy method for objective weighting

To eliminate subjective bias, this study applied the entropy method to assign weights to indicators. The steps are:

*Normalization* Raw data (e.g., tourist income) were normalized to a 0–1 scale.

*Entropy calculation* For each indicator *j*, entropy *E*_*j*_ was computed as:

1 $$\:{E}_{j}=-\frac{1}{{ln}n}\sum\:_{i=1}^{n}{p}_{ij}{ln}{p}_{ij}$$.

where$\:\:\:{P}_{ij}=\frac{{r}_{ij}}{\sum\:_{i=1}^{n}{r}_{ij}}$, *x*_*ij*_ is the normalized value of indicator *j* in region *i*, and *n* is the number of regions.

*Weight assignment* Weight $\:{\omega\:}_{j}$ for indicator *j* is:

2 $$\:{\omega\:}_{j}=\left(1-{E}_{j}\right)/{\varSigma\:}_{j=1}^{m}\left(1-{E}_{j}\right)$$.

Higher *w*_*j*_ indicates greater impact of *j* on TU.

##### State space method for integration

The TU index was derived using a state space model:

*Axis definition* Three axes represent urbanization, tourism, and eco-environment subsystem scores (calculated via entropy-weighted indicators).

*Vector calculation* Subsystem scores for each region were plotted as coordinates in the state space.

*Index calculation* The TU index is the Euclidean distance from the origin to the coordinate point:

3 $$\:TU=\sqrt{{S}_{urban}^{2}+{S}_{tourism}^{2}+{S}_{eco}^{2}}$$.

where *S*_*urban*_, *S*_*tourism*_, and *S*_eco_ are subsystem scores.

As a result of involving the calculation of tourism urbanization indicators for multiple regions and multiple years and considering the correlations between the various indicators in this study, the entropy method was utilized to eliminate subjective factors in assessing weight, thereby ensuring a more objective determination of the weights of each indicator. The detailed evaluation indicators and their corresponding weights are shown in Table 2.

**Table 2:** Tourism urbanization evaluation index system.

Subsystem	Indicator system (Unit)	Weight and attributes
Tourism Industry Subsystem	T1 Tourism Foreign Exchange Income/10,000 US Dollars	0.124 (+)
	T2 Domestic Tourism Income/100 Million CNY	0.071 (+)
	T3 Inbound Tourist Arrivals/10,000 Persons	0.109 (+)
	T4 Domestic Tourist Arrivals/10,000 Persons	0.073 (+)
	T5 Comprehensive Tourism Benefit Revenue/100 Million CNY	0.232 (+)
	T6% of Total Tourism Revenue to Gross Domestic Product (%)	0.024 (+)
Urbanization Subsystem	U1 Average Disposable Income per Urban Resident/CNY	0.025 (+)
	U2 Proportion of the Secondary and Tertiary Industry Output to GDP (%)	0.007 (+)
	U3 Urbanization rate (%)	0.015 (+)
	U4 Proportion of Employees in the Secondary and Tertiary Industries (%)	0.005 (+)
	U5 Number of Students in Regular Higher Education Institutions/Individuals	0.093 (+)
	U6 Per Capita Retail Sales of Consumer Goods/CNY	0.034 (+)
	U7 Number of Hospital Beds per 10,000 People/Bed	0.021 (+)
	U8 Per Capita Road Area(m^2^)	0.032 (+)
	U9 Proportion of Urban Built-up Area to Total Urban Land Area (%)	0.053 (+)
Eco-environment Subsystem	E1 Industrial Emissions of Sulfur Dioxide (t)	0.001 (-)
	E2 Industrial Wastewater Discharge (t)	0.003 (-)
	E3 Emissions of Industrial Particulate Matter (t)	0.004 (-)
	E4 Per Capita Park and Green Space Area (m^2^)	0.047 (+)
	E5 Greening Coverage Rate in Built-up Areas (%)	0.010 (+)
	E6 Comprehensive Utilization Rate of Industrial Waste (%)	0.009 (+)
	E7 Harmless Treatment Rate of Household Garbage (%)	0.008 (+)

The tourism urbanization evaluation index system comprises three subsystems: the tourism industry subsystem, the urbanization subsystem, and the eco-environment subsystem. Within this index system, T1, T2, T3, …, T6 represent the six indicators of the Tourism Industry Subsystem, while U1, U2, U3, …, U9 denote the nine indicators of the Urbanization Subsystem. Additionally, E1, E2, E3, …, E7 correspond to the seven indicators of the Eco-environment Subsystem. Positive indicators (denoted as “+”) are defined as those for which an increase in the quantity of the observed entities correlates with improved or more favorable outcomes; these are typically employed to assess positive developments. Conversely, negative indicators (denoted as “-“) are characterized by a direct relationship in which a higher count of the observed entities is associated with poorer or less favorable outcomes, and are generally utilized to evaluate adverse changes. A higher weight value indicates that the indicator exhibits considerable variability and has a more pronounced impact on the comprehensive evaluation.

#### Evaluation of ecosystem services

This study evaluated water yield, soil conservation, habitat quality, and carbon sequestration based on the Integrated Valuation of Ecosystem Services and Tradeoffs (InVEST) model in the YRB over the past 20 years. The detailed calculation process and formulas are shown in the supplementary material.

#### Measurement of coupling relationship between urbanization and ESs

For a meticulous analysis of the interplay between tourism urbanization and ESs, various functions of ESs and facets of tourism urbanization were considered as distinct subsystems within the broader frameworks of tourism urbanization and ESs (Fig. 3).

Spearman correlation analysis is suitable for assessing the relationship between two non-normally distributed continuous variables, while the coupled coordination degree (CCD) model is more suitable for describing interactions between two or more subsystems. Explore the relationship between tourism urbanization with its subsystem levels and four categories of ESs within Spearman correlation analysis in this study first. Next, the coupling coordination between tourism urbanization and ecosystem services (ESs) was analyzed utilizing the CCD model. The computational procedure involves: determining the coupling degree (CD) between ESs and tourism urbanization; calculating their comprehensive evaluation index, and examining the CCD based on the CD and comprehensive evaluation index, specific calculations are referred to references^36,45^.

This study utilized the CCD model to analyze the coupling coordination between the four categories of ecosystem services and the level of urbanization. The calculation formula is as follows:

5 $$Ci=2 \times \sqrt {\frac{{wi \times u}}{{{{\left( {wi+u} \right)}^2}}}}$$

6 $$T=\alpha wi+\beta u$$

7 $$D=\sqrt {C \times T}$$.

Ci represents the coupling degree between ecosystem services and the level of urbanization, where C∈[0, 1].

When C =1, the coupling degree is the highest, indicating a highly coordinated development of urbanization and ecosystem services, signifying their trend toward orderly development; conversely, they tend toward disorderly development.

Wi (i = 1, 2, 3, 4) and u are the comprehensive evaluation values of four categories of ecosystem services and the level of urbanization, respectively;

T is the comprehensive evaluation index of the ecosystem services subsystem and the urbanization subsystem;

α and β are weight values, with α = 0.5 and β = 0.5 based on previous research;

D represents the coupling coordination between ecosystem services and the level of urbanization.

Furthermore, the CCD is divided into the verge of disorder (0.0 ~ 0.2), passive coordination (0.2 ~ 0.4), low-level coordination (0.4 ~ 0.6), middle-level coordination (0.6–0.8), high-level coordination (0.8 ~ 1.0)^52^.

## Results

### Land use/land cover structure transformation

**Fig. 2:**
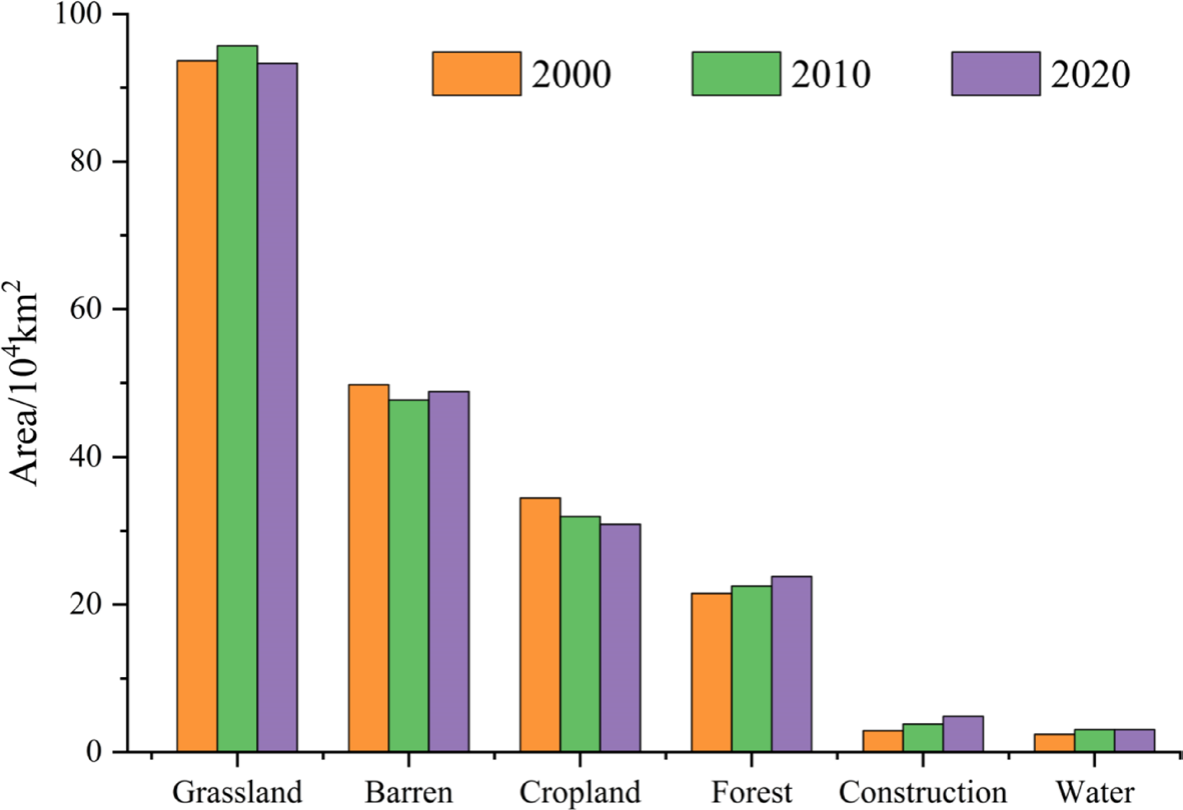
Changes of land use/land cover in the YRB from 2000 to 2020. *Note* Grassland is the predominant land use type, while areas allocated for Construction Land and Water Bodies are relatively minimal. Notably, there is a general trend of decrease in Grassland, Cropland, and Forest areas, with an increase in Construction over time.

Between 2000 and 2020, grassland and barren land were the dominant land cover types in the YRB, accounting for 46.02% (94.23 × 10^4^ km^2^) and 23.8% (48.73 × 10^4^ km^2^) of the total land area, respectively (Fig. 2). Cropland and forests followed, occupying 15.83% (32.42 × 10^4^ km^2^) and 22.62% (22.62 × 10^4^ km^2^). Notably, forest area increased by 2.31 × 10^4^ km^2^, whereas barren land and cropland decreased by 3.57 × 10^4^ km^2^ and 0.95 × 10^4^ km^2^, respectively.

**Fig. 3:**
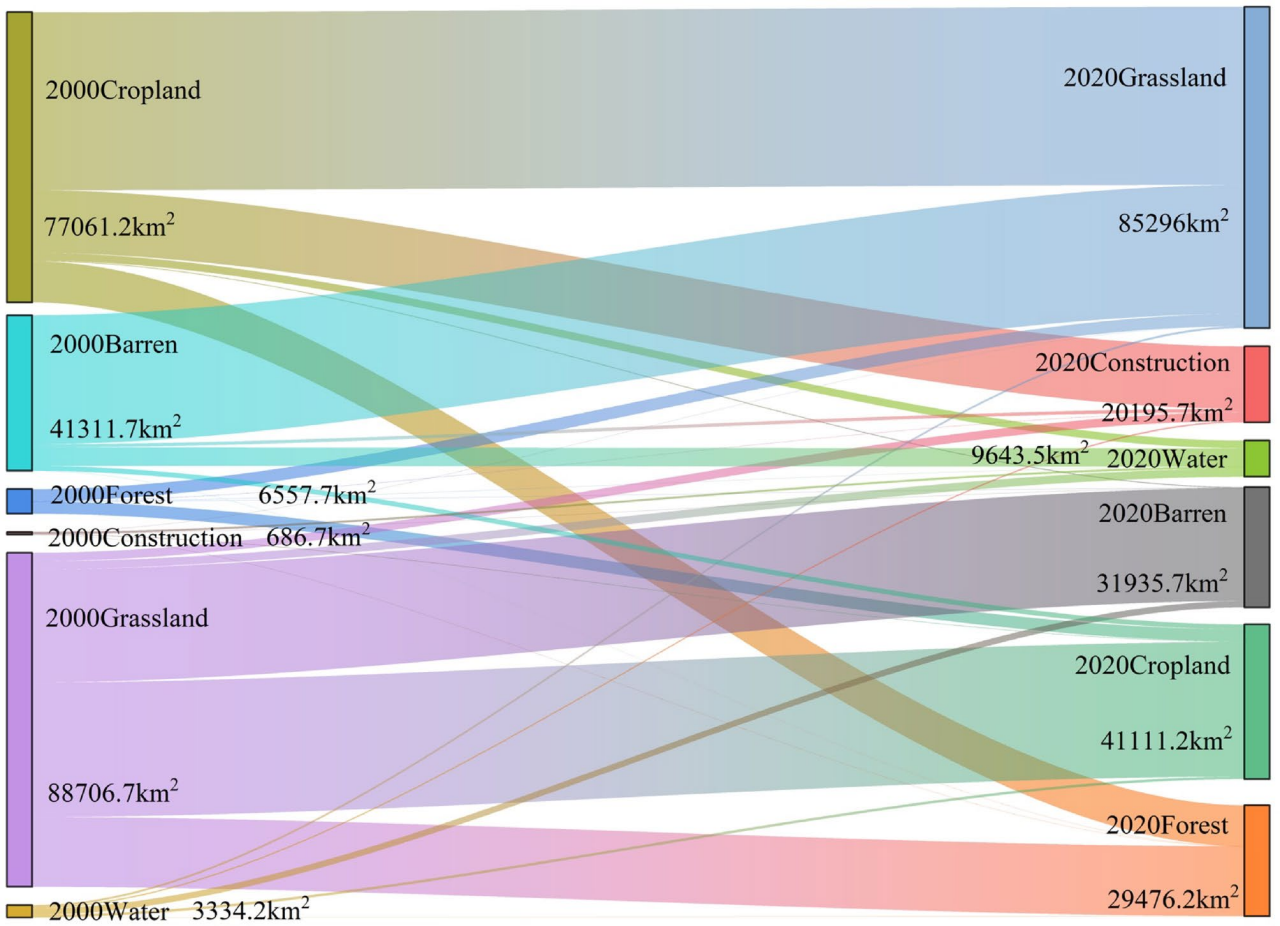
Sankey diagram depicting the transfer flows of various land use/land cover within the YRB between 2000 and 2020. *Note* The widths of the streams in the diagram represent the scale of area switching between different types of land use/land cover, illustrating the changes in land allocation for cropland, barren land, forests, construction sites, grassland, and water bodies in the preceding 20 years. Data derived from 2000–2020 land-use transition matrices.

Land use transition Sankey diagrams (Fig. 3) indicate that from 2000 to 2020, different land covers were converted into grassland, cropland, barren, forests, construction, and water bodies with areas of 85,296 km^2^ (39.18%), 41111.2 km^2^ (18.88%), 31935.7 km^2^ (14.67%), 29476.2 km^2^ (13.54%), 20195.7 km^2^ (9.27%), and 9643.5 km^2^ (4.43%), respectively. Among these, cropland was converted into grassland and forests with areas of 47,283 km^2^ and 10,891 km^2^, while grassland was converted into cropland, barren, and forests with areas of 35740.7 km^2^ and 18545.5 km^2^. Additionally, throughout this period, cropland, barren land, grassland, and water bodies transitioned into construction areas, encompassing 16619.2 km^2^, 2256.2 km^2^, 870 km^2^, and 364.2 km^2^, respectively. Furthermore, the predominant land use transitions among different land use types were from barren to grassland and from cropland to construction. In summary, between 2000 and 2020, the areas covered by forests and water bodies have increased, whereas the expansion of construction has contributed to the reduction of cropland and grassland areas.

### Spatiotemporal changes in tourism urbanization levels

**Fig. 4:**
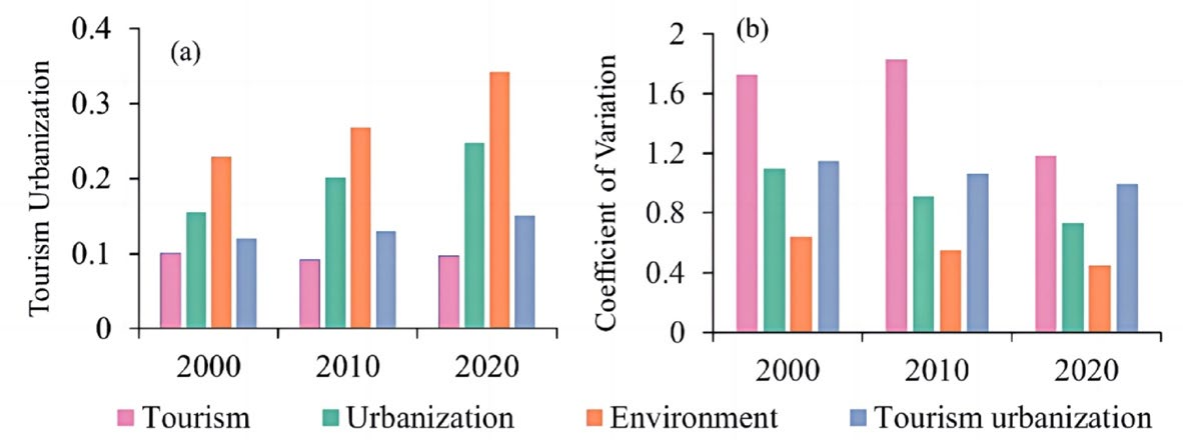
Temporal trends and variability of tourism urbanization subsystems in the YRB (2000–2020). *Note*(**a**) Development levels of tourism (TS), urbanization (US), and eco-environmental (ES) subsystems, showing a 37.59% increase in US and 33.19% in ES. (**b**) Coefficient of variation (CV) for TS, US, and comprehensive tourism urbanization (TU), with TU exhibiting the highest variability (CV decline: 13.4%).

The levels of tourism urbanization increased by 25% from 2000 to 2020, yet displayed significant spatial disparities (Fig. 4). The urbanization subsystem experienced a substantial surge of 37.59%, while the eco-environmental subsystem recorded a 33.19% increase, indicating improved ecological governance (Fig. 4a). An analysis of the coefficient of variation (Fig. 4b) reveals a reduction in development disparities across the YRB.

**Fig. 5:**
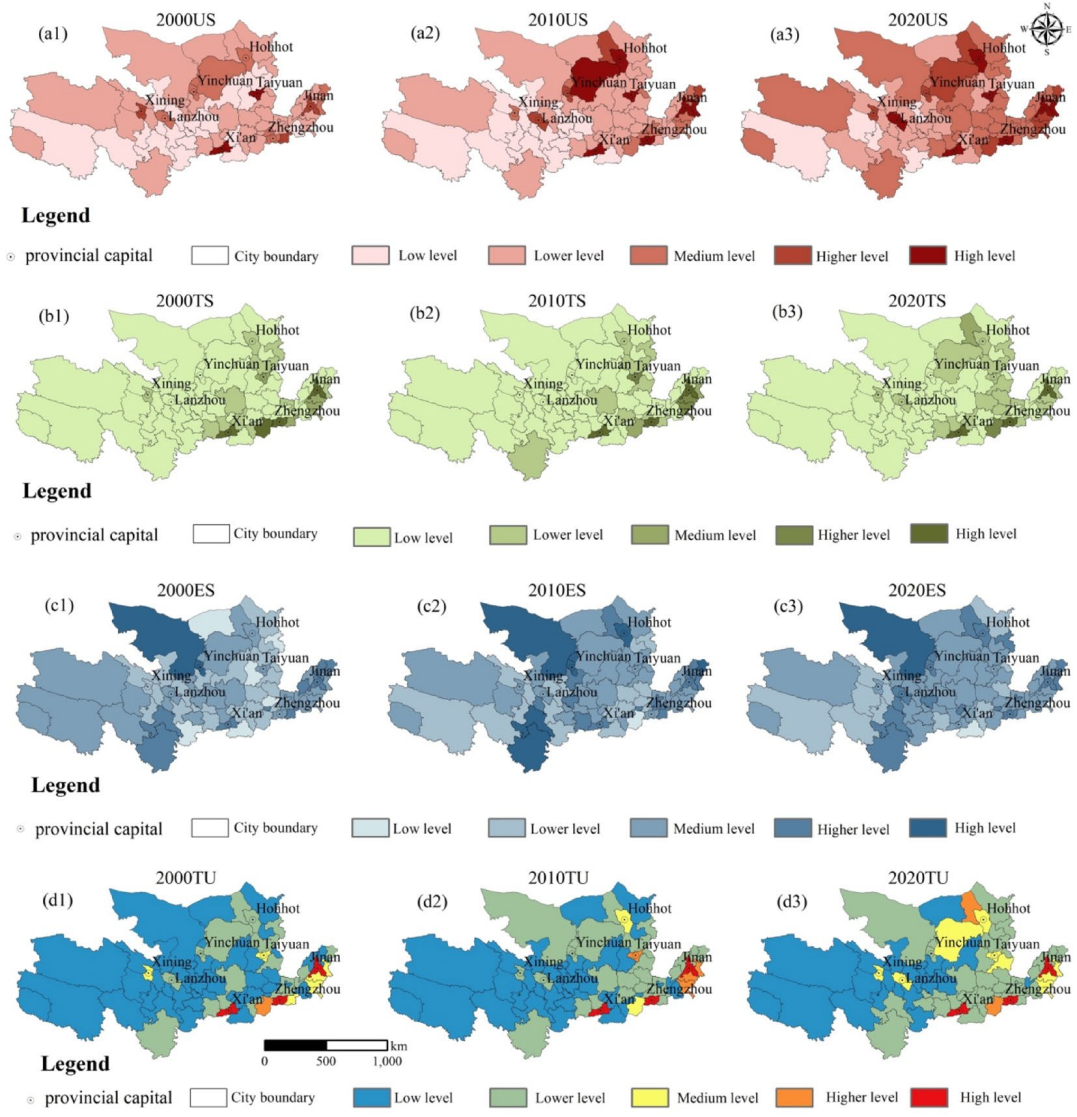
Spatiotemporal evolution of tourism urbanization subsystems in the YRB (2000–2020). *Note*(**a**) Urbanization subsystem (US) levels. (**b**) Tourism subsystem (TS) levels. (**c**) Eco-environmental subsystem (ES) levels. (**d**) Comprehensive tourism urbanization (TU) levels. Color thresholds: Low (< 0.2), Lower (0.2–0.4), Medium (0.4–0.6), High (0.6–0.8), Higher (> 0.8). Provincial capitals and city boundaries are marked for spatial reference. All graphs presented in this study were created by the author using ArcGIS 10.8, developed by the Environmental Systems Research Institute (ESRI) (https://www.esri.com/).

Spatially, tourism urbanization exhibited an ‘east-high, west-low’ distribution pattern (Fig. 5), with major growth observed in Shandong Peninsula, Central Plains, and Guanzhong Plain city clusters, while upstream regions such as LanXi City Cluster exhibited lower levels of development (Fig. 5a). The tourism subsystem (TS) levels showed a similar trend, expanding significantly in the eastern and central YRB, while remaining stagnant in northwestern regions (Fig. 5b). The eco-environmental subsystem (ES) levels followed an opposite gradient, decreasing from the periphery towards the center (Fig. 5c). Comprehensive tourism urbanization (TU) levels also exhibited spatial heterogeneity, with high values concentrated in urban centers and declining towards rural peripheries (Fig. 5d).

### Spatiotemporal variation characteristics of ESs

**Fig. 6:**
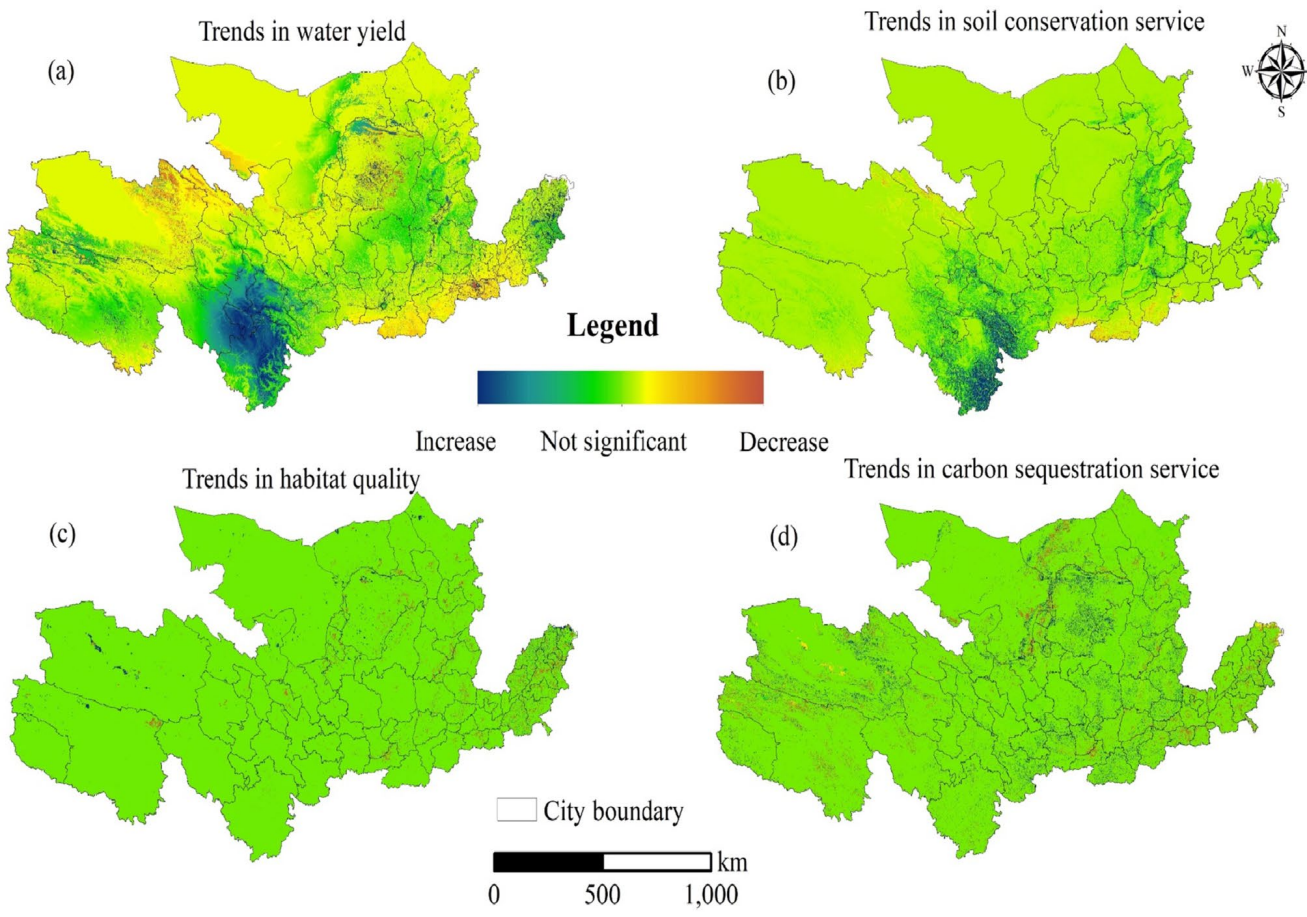
Spatiotemporal Trends in Ecosystem Service (WY, SC, HQ, CS) Annualized Change Rates (2000–2020). *Note*(**a**) The spatial distribution of Water Yield (WY) shows in 2000, 2010, and 2020. (**b**) The spatial distribution of Soil Conservation (SC). (**c**) The spatial distribution of Habitat Quality (HQ). (**d**) The spatial distribution of Carbon Sequestration (CS). Each map provides a visual representation of the respective ecosystem service distribution within the YRB, with the gradient scales indicating the intensity or quantity of ESs provided. City boundaries are marked to delineate the scope of service distribution. All graphs presented in this study were created by the author using ArcGIS 10.8, developed by the Environmental Systems Research Institute (ESRI) (https://www.esri.com/). *Trends here represent spatially explicit annualized change rates calculated as ΔES/year = (ES_2020_ – ES_2000_) / 20, where positive/negative values indicate increasing/decreasing trajectories, respectively. Significance testing (*p* < 0.05) was applied to validate trend robustness.

**Fig. 7:**
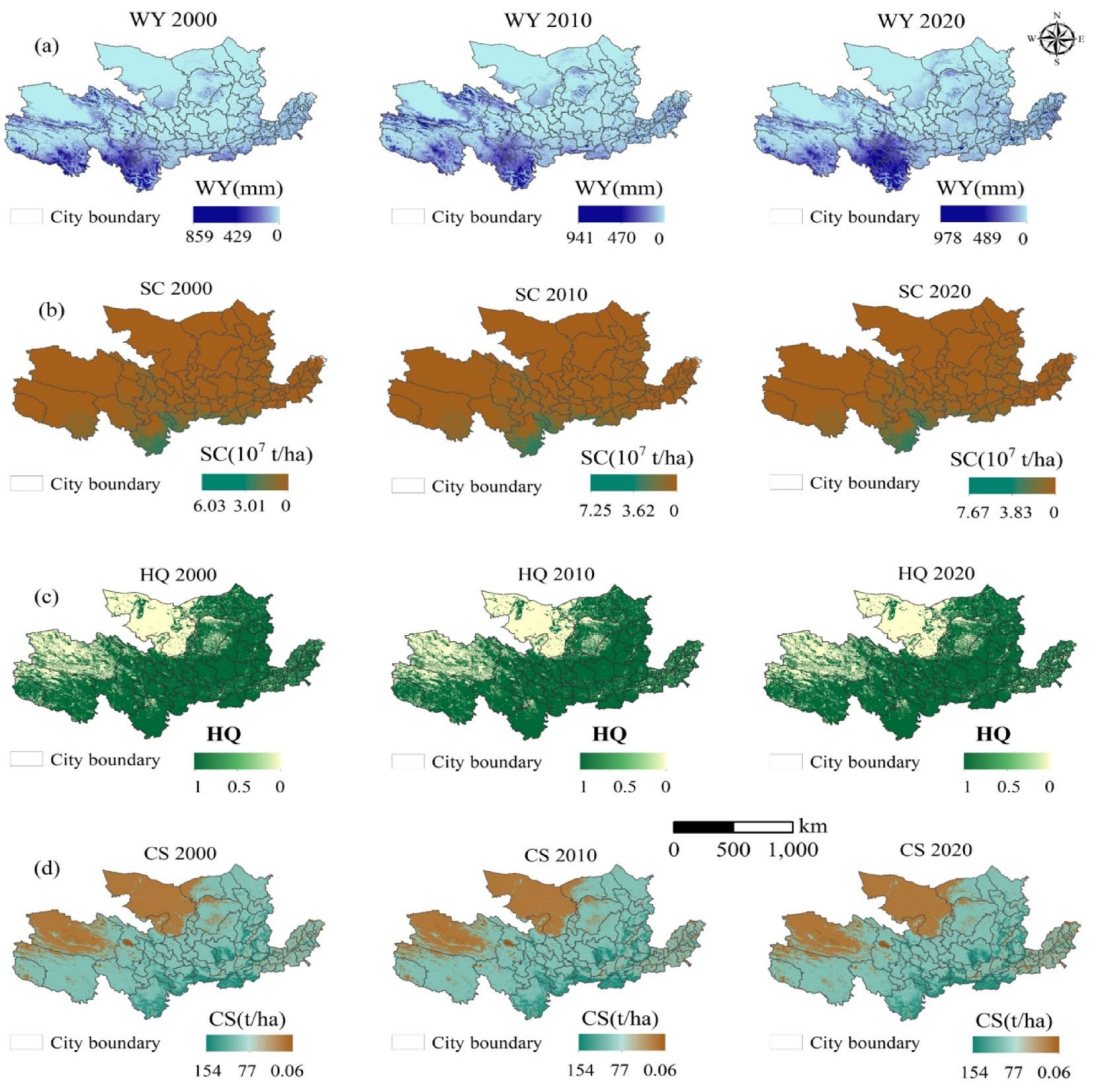
Trends of ecosystem services (WY, SC, HQ, CS) in the YRB between 2000 and 2020. *Note*(**a**) Shows areas with significant trends in Water Yield (WY) (Moran’s I > 0.35). (**b**) Depicts significant trends in Soil Conservation (SC) (Moran’s I > 0.16). (**c**) Illustrates areas with notable trends in Habitat Quality (HQ) (Moran’s I > 0.42). (**d**) Highlights regions with significant trends in Carbon Sequestration (CS) (Moran’s I > 0.28). The legend indicates areas of increase, no significant change, and decrease, with city boundaries provided for spatial context. All graphs presented in this study were created by the author using ArcGIS 10.8, developed by the Environmental Systems Research Institute (ESRI) (https://www.esri.com/).

Ecosystem services (ESs) displayed heterogeneous trends (Table 3; Figs. 6 and 7). Between 2000 and 2020, water yield (WY) and soil conservation (SC) increased by 64.95% and 23.26%, respectively (Table 3). In contrast, habitat quality (HQ) remained stable, while carbon sequestration (CS) exhibited minor fluctuations.

**Table 3:** Unit area of water yield (WY), soil conservation (SC), habitat quality (HQ), and carbon sequestration (CS) in 2000, 2010, and 2020 in the YRB.

	WY (mm)	SC (10^7^t/ha)	HQ	CS (t/ha)
2000	39.06	3.01	0.71	81.19
2010	49.66	3.39	0.71	81.54
2020	64.43	3.71	0.70	80.87

High-value areas for water yield (WY) and soil conservation (SC) were predominantly located in the Sanjiangyuan National Park, Qilian Mountains, and Wei River Basin, whereas low-value areas were identified in the North China Plain and the Loess Plateau (Fig. 6). The spatial distribution of WY (Fig. 6a) reveals an increasing gradient from east to west, while SC demonstrates a increasing trend from north to south (Fig. 6b). Patterns of habitat quality (HQ) and carbon sequestration (CS) were largely consistent, with high-value regions concentrated in forested and mountainous areas, including the Qilian Mountains and Sanjiangyuan National Park. Conversely, low-value zones were predominantly found in industrialized and agricultural regions (Fig. 6c).

Long-term trends (Fig. 7a) reveal that WY and SC significantly increased in reforested areas, whereas urbanized regions experienced declining CS levels, highlighting the trade-offs between conservation and development. The temporal evolution of SC (Fig. 7b) further demonstrates significant improvement in erosion control zones, particularly in the Loess Plateau and mountainous landscapes. However, HQ experienced a notable decline in urbanized regions, suggesting a deterioration in ecological integrity (Fig. 7c). Forest-dominated regions in the southwestern YRB have seen substantial carbon sequestration (CS) gains, consistent with reforestation policies and reduced deforestation rates. However, the North China Plain and industrial corridors have exhibited declining CS trends, likely due to cropland expansion and fossil fuel emissions. These spatial heterogeneities highlight the interplay between ecological restoration efforts and human-induced pressures in shaping ecosystem service trajectories across the YRB (Fig. 7d).

#### Spatial regression analysis

Spatial auto-correlation is commonly used to detect potential spatial dependence between observed attribute values within a region. Moran’s I is a spatial autocorrelation statistic that quantifies the degree to which similar or dissimilar values cluster geographically. It answers: (1) Are high/low values of a variable (e.g., habitat quality) spatially clustered? (2) Is the observed spatial pattern random, dispersed, or aggregated? In this study, spatial auto-correlation of tourism urbanization and ESs in the YRB is measured using *GeoDa 1.20*, the specific calculation process and equation refer to references^53^. All graphs presented in this study were created by the author using ArcGIS 10.8, developed by the Environmental Systems Research Institute (ESRI) (https://www.esri.com/).

When I > 0: Positive spatial autocorrelation (similar values cluster; e.g., high habitat quality regions adjacent to other high-value areas). I < 0: Negative spatial autocorrelation (dissimilar values cluster; e.g., high-value areas surrounded by low-value zones). I ≈ 0: No spatial autocorrelation (random distribution).

The Global Moran’s I was calculated as:

4 $$I=\frac{n}{{{S_0}}} \cdot \frac{{\sum\limits_{{i=1}}^{n} {\sum\limits_{{j=1}}^{n} {{w_{ij}}\left( {{x_i} - \bar {x}} \right)\left( {{x_j} - \bar {x}} \right)} } }}{{\sum\limits_{{i=1}}^{n} {{{\left( {{x_i} - \bar {x}} \right)}^2}} }}$$.

Where: *n*: Number of spatial units. *w*_*ij*_ : Spatial weight between units *i* and *j*(queen contiguity weights used). *S*_0_=$\sum\limits_{{i=1}}^{n} {\sum\limits_{{j=1}}^{n} {{w_{ij}}} }$: Sum of all spatial weights. *x*_*i*_: Observed value at unit *i*. $\bar {x}$: Mean of all observed values.

### Interactive relationship between ESs and tourism urbanization

#### Correlation between tourism urbanization and ESs

**Fig. 8:**
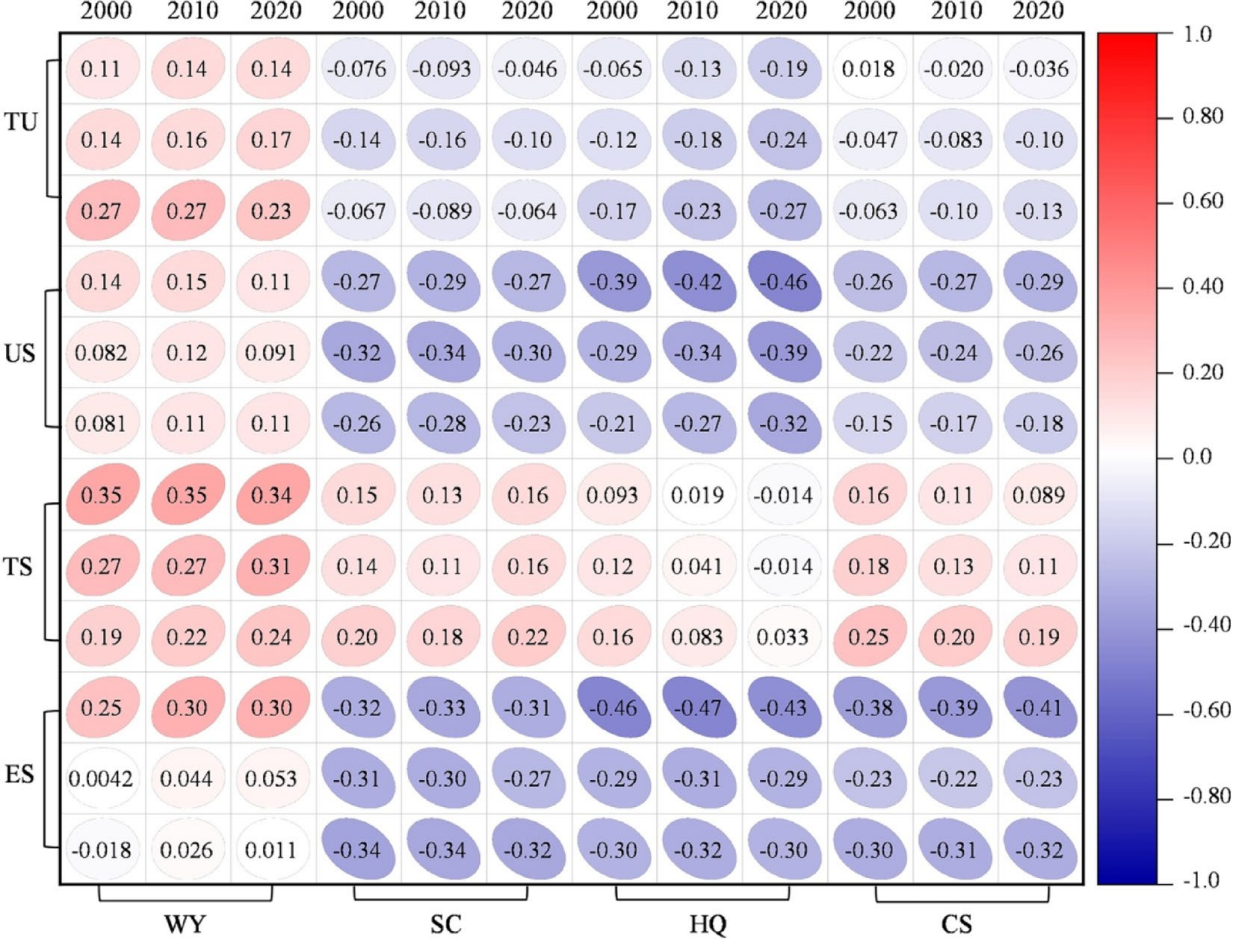
Correlation matrix showing the relationship between the tourism urbanization level of the Yellow River Basin and ecosystem services from 2000 to 2020. *Note* The ecosystem services include Water Yield (WY), Soil Conservation (SC), Habitat Quality (HQ), and Carbon Sequestration (CS). The subsystems analyzed are Urbanization Services (US), Tourism Services (TS), Environment Services (ES), and Tourism Urbanization (TU). The correlation coefficient values are shown by a color gradient, where red denotes a positive correlation and blue denotes a negative correlation.

Spearman’s correlation analysis (Fig. 8) showed a negative correlation between tourism urbanization and HQ, SC, and CS, whereas WY exhibited a positive association. However, these negative effects weakened over time, suggesting an evolving balance between tourism growth and ecosystem sustainability.

#### Analysis of CCD between ESs and tourism urbanization

**Fig. 9:**
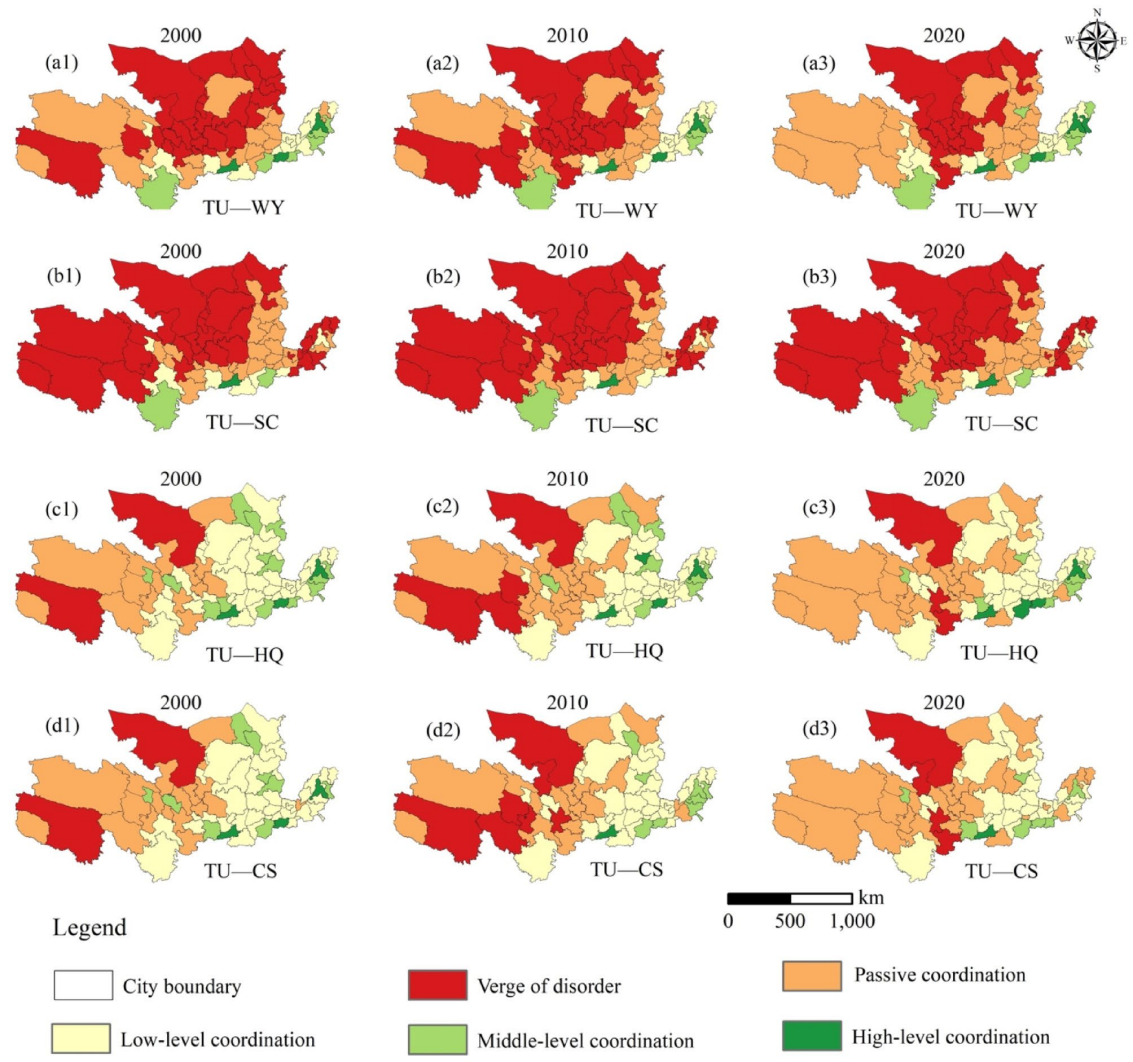
City-scale spatial pattern of the coupling coordination degree involving tourism urbanization and ESs in 2000, 2010, and 2020. *Note*(**a**) Shows the coordination with Water Yield services (WY), (**b**) illustrates the coordination with Soil Conservation services (SC), (**c**) depicts the coordination with Habitat Quality (HQ), and (**d**) represents the coordination with Carbon Sequestration services (CS). Coordination levels: red = severely imbalanced (CCD < 0.3), orange = marginally coordinated (0.3–0.5), light green = moderately coordinated (0.5–0.7), dark green = highly coordinated (> 0.7). The maps include city boundaries for geographical context. All graphs presented in this study were created by the author using ArcGIS 10.8, developed by the Environmental Systems Research Institute (ESRI) (https://www.esri.com/).

Between 2000 and 2020, the coupling coordination degree (CCD) between tourism urbanization and ecosystem services (ESs) in the Yellow River Basin (YRB) showed a declining trend (Fig. 9).

Notably, the CCD between tourism urbanization and water yield (WY) exhibited the least decline, indicating that water resources have demonstrated relative resilience to the impacts of urbanization (Fig. 9a). In contrast, soil conservation (SC) displayed the most significant imbalance, with approximately 86.4% of the YRB experiencing a decrease in CCD values, particularly in the Loess Plateau and North China Plain, where soil erosion and land degradation have intensified (Fig. 9b). Spatially, regions with high coordination were identified in the Shandong Peninsula and Central Plains, while low-coordination zones were predominantly located in the Loess Plateau and North China Plain (Fig. 9c). Carbon sequestration (CS) experienced moderate declines in CCD, primarily in industrialized areas where land conversion and emissions have diminished ecological resilience (Fig. 9d).

## Discussion

### Spatial imbalances and developmental trajectories in tourism urbanization

The Yellow River Basin (YRB) exhibits pronounced spatial imbalances in tourism urbanization, characterized by an “east-high, west-low” gradient, aligning with regional economic disparities and infrastructure investments (Fig. 5d)^14,54^. Urban clusters such as the Shandong Peninsula demonstrated rapid expansion, reaching urbanization indices above 0.34 by 2020, whereas upstream regions like LanXi City Cluster stagnated with indices below 0.15. This pattern, partially driven by policy-directed resource allocation, reshaped urban-rural development gaps and land-use transitions, with 16,619.2 km^2^ of cropland converted into construction areas (Fig. 3).

Tourism urbanization also revealed subsystem asynchronicity, a phenomenon previously noted in the Pearl River Delta’s industrial-ecological systems^55^. while urbanization and eco-environmental subsystems reached over 68% and 72% of their development potential, the tourism subsystem lagged at 43% efficiency, primarily due to its limited integration with ecosystem services (ESs) (Fig. 4a)^14,54^. This discrepancy underscores the YRB’s unique challenge in reconciling tourism growth with ES conservation. It resulted in deteriorating coupling coordination degrees (CCD), particularly for soil conservation (SC) and habitat quality (HQ), which declined by 9.3% and 11.6%, respectively, in fragile landscapes such as the Loess Plateau (CCD < 0.41) (Fig. 9)^54^. Such findings highlight how rapid urbanization can drive ES degradation, necessitating a re-evaluation of land-use policies to mitigate long-term ecological trade-offs.

Recent trends (2018–2022) suggest that certain regions may transition to more balanced urbanization. For example, the Guanzhong Plain cluster (CCD = 0.58 ± 0.07) is poised to reach high-intensity urbanization by 2025, with tourism contributing to 37.6% of system momentum^54^. Similarly, Lanzhou’s annual urban growth rate exceeding 4.2% indicates a potential diffusion of development benefits beyond core tourism hubs. However, reliance on municipal-scale datasets limits the ability to capture fine-scale ecological responses, suggesting a need for higher-resolution spatial analysis in future studies.

### Ecosystem services (ESs) dynamics and multiscale drivers

Spatial heterogeneity in ecosystem services (ESs) across the YRB is influenced by both natural and anthropogenic factors. High-value ES clusters, such as Sanjiangyuan National Park, displayed robust water yield (WY: 64.43 mm by 2020) and soil conservation (SC: 4.15 × 10⁷ t/ha), primarily driven by dense forest (22.62%) and grassland (46.02%) coverage (Fig. 2; Table 3)^55^. In contrast, low-value ES regions, such as the North China Plain, experienced habitat degradation, with HQ declining to 0.70 in 2020 due to rapid urban expansion (construction land increased by 1.95 × 10^4^ km^2^, Fig. 3)^55^.

Land-use transitions played a pivotal role in ES variation. Forest expansion (+ 2.31 × 10^4^ km^2^, Fig. 3) enhanced carbon sequestration (CS: 80.87 t/ha, Table 3) and improved hydrological regulation. Meanwhile, cropland-to-construction conversions (16,619.2 km^2^, Fig. 3) in downstream hubs resulted in reduced ES stability, particularly in soil conservation and habitat quality. While erosion control measures in the Loess Plateau improved SC, they failed to compensate for HQ losses in urban peripheries (Fig. 7c)^56^.

These findings reinforce the spatial dependencies of ESs, consistent with studies in other regions that emphasize vegetation coverage as a key driver of ES provision^57,58^. However, the YRB’s HQ degradation in urban peripheries diverges from the stable HQ patterns observed in the EU’s Natura 2000 protected areas^59^, likely due to weaker regulatory enforcement in balancing tourism growth and habitat conservation. Additionally, the study’s exclusion of cultural services (e.g., aesthetic value) contrasts with integrated ES assessments^60^, suggesting future studies should adopt multi-dimensional frameworks to capture tourism’s full socio-ecological impact.

### Coupling coordination between tourism urbanization and ecosystem services

The interaction between tourism urbanization and ESs exhibits complex trade-offs and evolving synergies. Initially, rapid construction expansion (1.95 × 10^4^ km^2^, Fig. 3) in downstream regions (e.g., Shandong Peninsula) contributed to ES degradation, with CCD values for carbon sequestration (CS) declining by 12.96% (Fig. 9d)^19^. However, over time, these trade-offs became less pronounced, reflecting adaptive governance measures that improved urban ecological resilience.

The CCD analysis (Fig. 9d) reveals that while water yield (WY) remained relatively stable, soil conservation (SC) exhibited the greatest imbalance, particularly in the Loess Plateau and North China Plain, where urbanization pressures intensified soil degradation^54^. The Guanzhong Plain’s CCD (0.58 ± 0.07, Fig. 9) indicates a threshold beyond which urbanization may transition towards synergy with ES conservation, contingent on green infrastructure investments and stricter land-use regulations^2,19^.

Despite these insights, this study’s focus on municipal-scale data may obscure localized ecosystem vulnerabilities. Moran’s I spatial dependencies (HQ > 0.42, Fig. 7c) suggest that fine-scale variations within ES provision remain underexplored. Future research should integrate high-resolution geospatial modeling to capture neighborhood-level ES dynamics, particularly in wetland and riparian ecosystems^61^.

### Policy implications and sustainable governance strategies

Given the observed trade-offs between tourism urbanization and ecosystem services (ESs), targeted policy interventions are essential for achieving sustainable development. This study identifies several key strategies: (1) Green Infrastructure Development. In high-urbanization zones, such as the Shandong Peninsula and Guanzhong Plain, expanding urban green spaces and implementing wetland restoration projects could mitigate declines in ESs, particularly in cultural services (CS) and habitat quality (HQ)^61^. (2) Differentiated Land-Use Regulations. In areas with medium coordination, such as the Loess Plateau, adaptive zoning policies that restrict construction in high-erosion risk regions could enhance ES resilience while supporting tourism growth^54^. (3) Ecological Compensation Mechanisms. Implementing regional ecological compensation programs could balance resource demands, ensuring that the degradation of ESs driven by urbanization is offset by conservation investments^10^. By aligning these strategies with global sustainability initiatives, such as the UN Decade on Ecosystem Restoration, the Yellow River Basin (YRB) provides a valuable case study for integrating tourism-driven growth with ecological stewardship, offering insights for other fragile basins worldwide^62^.

## Conclusion

The spatiotemporal interplay between tourism urbanization and ecosystem services (ESs) in the Yellow River Basin (YRB) is characterized by pronounced spatial polarization and declining coupling coordination degrees (CCD), as revealed through integrated entropy-InVEST modeling (Figs. 2, 3, 4, 5, 6, 7, 8 and 9). Key findings include:

(1) *Asymmetric land-use transitions* Construction land expanded by 1.95 × 10^4^ km^2^ (2000–2020) at the expense of cropland and grassland (Fig. 3), driving a 12.96% CCD decline between tourism urbanization and carbon sequestration (Fig. 9d).

(2) *Subsystem asynchronicity* Urbanization and eco-environmental subsystems achieved 68% and 72% of their potential, respectively, while the tourism subsystem lagged at 43% (Fig. 4a), reflecting policy-driven imbalances in resource allocation.

(3) *Transitional zone resilience* Despite intensive urbanization, the Guanzhong Plain maintained stable CCD (0.58 ± 0.07; Fig. 9d), suggesting thresholds for sustainable transitions in ecologically vulnerable regions.

These findings extend coupling coordination theory by identifying spatial heterogeneity as a critical mediator of tourism-ES interactions, contrasting with Mediterranean models where policy inertia perpetuates imbalances^2,19^. The proposed three-tier governance framework—prioritizing green infrastructure in high-coordination zones (CCD ≥ 0.55), ecological compensation in transitional areas (0.35 ≤ CCD < 0.55), and slope-based restrictions in fragile regions (CCD < 0.35) —provides a replicable model for balancing growth and ecology in global river basins. For instance, the Shandong Peninsula’s wetland restoration strategy mirrors the Mississippi River Basin’s floodplain management^63^, while the Loess Plateau’s slope restrictions align with Nepal’s landslide mitigation policies^64^. This framework’s emphasis on tourism-specific pressures complements the EU’s Water Framework Directive^65^, which focuses on industrial and agricultural impacts, thereby addressing a critical gap in basin-scale ES management.

Several limitations warrant attention. First, municipal-scale data, while critical for regional policy alignment, may spatially average fine-grained ES variations (e.g., wetland fragmentation in the Yellow River Delta)^66^, leading to underestimated local trade-offs—a issue similarly noted in the Mekong Basin’s ES assessments^67^. Second, the exclusion of cultural services (e.g., recreational aesthetics) limits holistic CCD evaluation, contrasting with integrated frameworks like the UK National Ecosystem Assessment^68^. Third, our model’s assumption of linear urbanization-ES interactions diverges from threshold-based approaches in socio-ecological resilience studies^69^, potentially oversimplifying feedbacks in fragile landscapes. Future work should integrate high-resolution remote sensing and dynamic system models to resolve these gaps.

## Electronic supplementary material

Below is the link to the electronic supplementary material.

Supplementary Material 1

## Data Availability

The datasets generated and/or analyzed during the current study are not publicly available at this time as they form an integral part of ongoing research investigations. However, the data are available from the corresponding author (Jingbo Zhou) upon reasonable request.
