# Supplementary material for: Evaluation of coupling coordination degree between tourism urbanization and ecosystem services in urban agglomerations in the yellow river basin
Source: Sci Rep. 2025 Jul 1;15:22427. doi: 10.1038/s41598-025-05455-6 (PMC12215424; doi:10.1038/s41598-025-05455-6)
Supplement: Supplementary file 1 — Supplementary Material 1 [file 41598_2025_5455_MOESM1_ESM.docx]

**Supplementary materials**

The InVEST model (Integrated Valuation of Ecosystem Services and Tradeoffs) is a system of models that can be used to assess the quantity and economic value of ecosystem services, supporting ecosystem management and decision-making. It includes assessment models for three categories of ecosystems: terrestrial, freshwater, and marine. In this paper, we conducted an assessment of water yield, soil retention services, habitat quality, and carbon sequestration services in the Yellow River basin using InVEST 3.10.0 (Sharp et al., 2015).

(https://naturalcapitalproject.stanford.edu/software/invest)

**1.1 Water Yield**

Calculate the annual water yield in the Yellow River basin using the Water Yield module of the InVEST model (Xu et al.,2022).

Where *Y**_jx_* is the annual water yield(mm)；*Px* is the annual precipitation(mm)；*AET_x_* is the annual Potential evaporation (mm)。

**1.2 Soil Retention**

Calculate soil retention using the Sediment Delivery Ratio module of the InVEST model with the following formula (Fu et al.,2011):

*SEDRETx = RKLSx - USLEx*

*Where SEDRETx* is the soil retention of grid cell *x*. *RKLSx* and *USLEx* represent the potential soil erosion (*t*), actual soil erosion (*t*), and sediment retention (*t*) intercepted by grid cell *x*, respectively.

*RKLSx = Rx × Kx × LSx*

*USLEx = Rx × Kx × LSx × Cx × Px*

Where *R_x_* represents rainfall erosivity [MJ·mm/(hm2·h·a)]; *K_x_* stands for soil erodibility; *LS_x_* denotes slope length-slope factor; *C_x_* represents vegetation cover factor; and *P_x_* is the management factor.

**1.3 Habitat quality**

Habitat quality refers to the capacity of an ecosystem to provide a suitable environment for the continuous survival of individuals and populations, and its level can reflect the degree of habitat fragmentation in the region and the resistance to habitat degradation (Moreira et al.,2018).

In the formula, *Q_xj_* represents the habitat quality of grid *x* in land use type *j*; *H_j_* represents the habitat suitability of land use type *j*; represents the level of stress experienced by grid *x* in land use type *j*; *z* is a normalization constant; and *k* is the scaling constant.

**1.4 Carbon sequestration**

This study calculates ecosystem carbon sequestration by multiplying the average carbon density of four carbon pools (aboveground biomass, belowground biomass, soil organic matter, and litter) from the InVEST model by the area of different land use/cover types (Garrastazú et al,2015). The specific calculation formula is as follows:

*C*_total = *C*_above + *C*_below + *C_*soil + *C*_dead

In the equation, *C*_total, *C*_above, *C*_below, *C*_soil, and *C*_dead represent total carbon stock, aboveground, belowground, soil, and litter carbon stock (t), respectively.

**2.1 Land Use Transfer Matrix**

Table S1 Land Use Transfer Matrix (km^2^) The matrix displays the transitions between different land use categories over 20 years in the Yellow River Basin. Categories include Barren, Construction, Cropland, Forest, Grassland, and Water. The rows represent the land use status in the year 2000, while the columns correspond to the status in 2020. Diagonal elements represent the area that remained unchanged, whereas off-diagonal elements indicate the area transferred from one land use type to another. The totals provide a summation of areas for each land use category at the beginning and end of the period, as well as the aggregate changes.

Table S1 Land Use Transfer Matrix (km^2^)

| 2020  2000 | Barren | Construction | Cropland | Forest | Grassland | Water | Total |
| --- | --- | --- | --- | --- | --- | --- | --- |
| Barren | - | 870 | 1314 | 1.2 | 34234.5 | 4892 | 41311.7 |
| Construction | 5 | - | 136 | 0.2 | 5.7 | 539.7 | 686.7 |
| Cropland | 221.5 | 16619.2 | - | 10891 | 47283 | 2046.5 | 77061.2 |
| Forest | 0.2 | 86 | 3241.7 | - | 3227.2 | 2.5 | 6557.7 |
| Grassland | 30001.5 | 2256.2 | 35740.7 | 18545.5 | - | 2162.7 | 88706.7 |
| Water | 1707.5 | 364.2 | 678.7 | 38.2 | 545.5 | - | 3334.2 |
| Total | 31935.7 | 20195.7 | 41111.2 | 29476.2 | 85296 | 9643.5 | 217658.5 |

**Reference**

1. Fu, B. J., Liu, Y., Lü, Y. H., He, C. S., Zeng, Y., & Wu, B. F. Assessing the soil erosion control service of ecosystems change in the Loess Plateau of China. *Ecol. Complex.*, **8** (4), 284-293(2011). <https://doi.org/10.1016/j.ecocom.2011.07.003>
2. Garrastazú, M. C., Mendonça, S. D., Horokoski, T. T., Cardoso, D. J., Rosot, M. A. D., Nimmo, E. R., & Lacerda, A. E. B. d. Carbon sequestration and riparian zones: assessing the impacts of changing regulatory practices in Southern Brazil. *Land Use Pol.*, **42** (0), 329-339(2015). <https://doi.org/10.1016/j.landusepol.2014.08.003>
3. Moreira, M., Fonseca, C., Vergílio, M., Calado, H., & Gil, A. Spatial assessment of habitat conservation status in a Macaronesian island based on the InVEST model: a case study of Pico Island (Azores, Portugal). *Land Use Pol.,* **78** (0), 637-649(2018). <https://doi.org/10.1016/j.landusepol.2018.07.015>
4. Sharp, R., Tallis, H.T., Ricketts, T., Guerry, A.D., Wood, S.A., Chapin-Kramer, R., Nelson, E., Ennaanay, D., Wolny, S., Olwero, N., Vigerstol, K., Pennington, D., Mendoza, G., Aukema, J., Foster, J., Forrest, J., Cameron, D., Arkema, K., Lonsdorf, E., Kennedy, C., Verutes, G., Kim, C.K., Guannel, G., Papenfus, M., Toft, J., Marsik, M., Bernhardt, J., Grifn, R., Gowinski, K., Chaumont, N., Perelman, A., Lacayo, M., Mandle, L., Hamel, P., Vogl, A.L., Rogers, L., Bierbower, W., 2015. InVEST 3.2.0 User’s Guide. The Natural Capital Project. Stanford University, University of Minnesota, The Nature Conservancy, and World Wildlife Fund. <https://naturalcapitalproject.stanford.edu/software/invest>
5. Xu, H. J., Zhao, C. Y., Wang, X. P., Chen, S. Y., Shan, S. Y., Chen, T., Qi, X. L. Spatial differentiation of determinants for water conservation dynamics in a dryland mountain. *J. Clean. Prod.*, **362** (0), 132574-132574(2022). <https://doi.org/10.1016/j.jclepro.2022.132574>
